# Supplementary material for: Silk Peptide Ameliorates Sarcopenia through the Regulation of Akt/mTOR/FoxO3a Signaling Pathways and the Inhibition of Low-Grade Chronic Inflammation in Aged Mice
Source: Cells. 2023 Sep 12;12(18):2257. doi: 10.3390/cells12182257 (PMC10527450; doi:10.3390/cells12182257)
Supplement: Supplementary file 1 [file cells-12-02257-s001.zip › Supplymentary data S1.pdf]

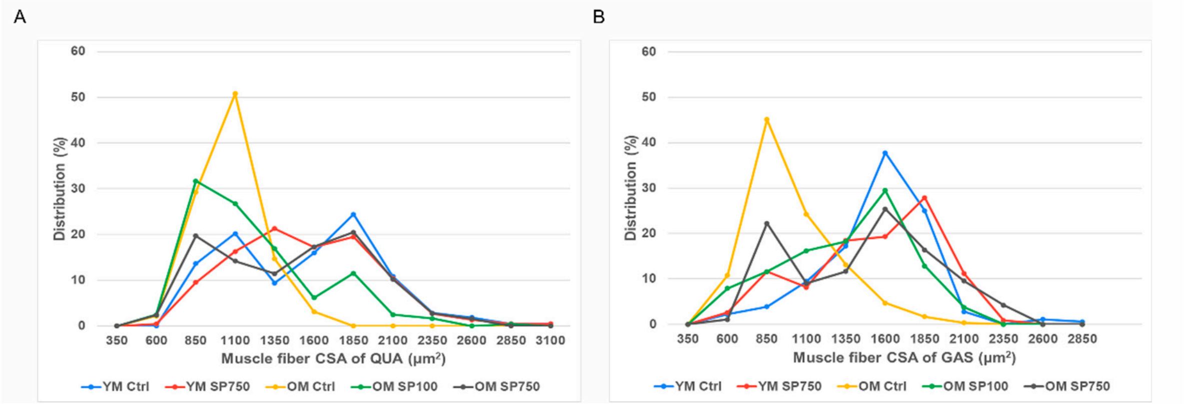

Figure S1. Distribution of muscle fiber cross-sectional areas (CAS) of (A) *quadriceps* (QUA) and (B) *gastrocnemius* (GAS) muscles.
